# An Arthroscopic Robotic System for Meniscoplasty with Autonomous Operation Ability

**DOI:** 10.3390/bioengineering12050539

**Published:** 2025-05-17

**Authors:** Zijun Zhang, Yijun Zhao, Baoliang Zhao, Gang Yu, Peng Zhang, Qiong Wang, Xiaojun Yang

**Affiliations:** 1 School of Robotics and Advanced Manufacturing, Harbin Institute of Technology, Shenzhen 518055, China; 23s153091@stu.hit.edu.cn (Z.Z.); 15635023789@163.com (Y.Z.); yangxiaojun@hit.edu.cn (X.Y.); 2 Shenzhen Institute of Advanced Technology, Chinese Academy of Sciences, Shenzhen 518055, China; bl.zhao@siat.ac.cn (B.Z.); wangqiong@siat.ac.cn (Q.W.)

**Keywords:** arthroscopic robot, point cloud registration, path-planning, remote center of motion

## Abstract

Meniscoplasty is a common surgical procedure used to treat meniscus tears. During the operation, there are often key challenges such as a limited visual field, a narrow operating space, and difficulties in controlling the resection range. Therefore, this study developed an arthroscopic robotic system with the ability of autonomous meniscus resection to achieve better surgical outcomes. To address the issue of limited visual fields during the operation, this study used the preoperative and intraoperative meniscus point cloud images for surgical navigation and proposed a novel cross-modal point cloud registration framework. After the registration was completed, the robotic system automatically generated a resection path that could maintain the crescent shape of the remaining meniscus based on the improved Rapidly Exploring Random Tree (RRT) path-planning algorithm in this study. Meanwhile, the Remote Center of Motion (RCM) constraint was introduced during the movement of the robot to enhance safety. In this study, the mean squared error of the preoperative–intraoperative meniscus point cloud registration was only 0.1964 mm^2^, which meets the surgical accuracy requirements. We conducted experiments to validate the autonomous operation capabilities of the robot. The robot successfully completed motion-planning and autonomous implementation, thus demonstrating the reliability of the robotic system.

## 1. Introduction

Clinically, knee meniscus tears account for approximately 12–14% of orthopedic knee injuries. Such injuries mainly occur in athletes and middle-aged and elderly populations [1]. For severe meniscus tears, meniscoplasty is commonly employed clinically. The surgery aims to preserve the healthy meniscus structure of patients to the greatest extent and maintain the crescent shape of the meniscus, thereby avoiding the occurrence of complications such as abnormal lower-limb biomechanical axes and overloading of the cruciate ligaments [2]. Meniscoplasty exhibits remarkable efficacy for radial tears, horizontal tears, and complex injuries of the meniscus [3]. However, during the clinical implementation, surgeons often face challenges such as limited visual fields and difficulties in performing delicate operations in confined spaces [4]. Moreover, they need to frequently adjust the pose of surgical instruments to avoid damaging healthy tissues. In response to these issues, this study intends to develop an arthroscopic robotic system. By leveraging robotic-assisted surgery, the study aims to reduce surgical complexity and enhance surgical quality.

Arthroscopic meniscoplasty is a type of endoscopic surgery. Currently, numerous robotic systems have been applied in this kind of surgery. The da Vinci system [5], developed by Intuitive Surgical in the United States, consists of the following three main components: a console, robotic arms, and surgical instruments on the patient side [6]. Surgeons can flexibly control the movement of the endoscope and surgical instruments at the end of the robotic arms through the handles on the console and carry out delicate and stable operations by combining with the real-time images on the high-definition 3D display screen [7], thus improving the precision of the surgery. In 2010, Jens et al. [8] successfully performed hip arthroscopy on two human cadavers using the da Vinci system. Subsequently, in 2011, Murat et al. [9] achieved successful shoulder arthroscopy on human cadavers using the da Vinci system. A multitude of studies have validated the clinical feasibility of robot-assisted arthroscopy. The Senhance surgical robotic system [10,11] is a laparoscopic robotic system developed by TransEnterix in Italy. The main components and operation mode of this system are like those of the da Vinci system. However, it innovatively introduces eye-tracking technology to help doctors control the endoscope and surgical instruments through their line of sight, reducing the complexity of manual operation required in traditional surgeries. Li et al. [12] proposed a prototype of a two-arm robot-assisted arthroscopic surgical system. One of the arms is integrated with an impedance controller and a gravity compensation mechanism, which is used to control the pose of the arthroscope during the operation. The other arm incorporates a point-based VF generation algorithm, which is applied to control the pose of the surgical instruments during the operation. Wu et al. [13] proposed an autonomous robotic knee arthroscopy system in concept, which comprises steerable robotic tools, autonomous leg manipulators, miniature stereo cameras, and 3D/4D ultrasound imaging systems.

However, existing studies have all focused on how to use robotic technology to assist doctors in performing more dexterous and precise surgical actions. During the surgical process, doctors still need to operate throughout the whole process, and robotic systems lack the ability to autonomously complete surgical subtasks.

Currently, numerous research institutions have carried out a large amount of research on tasks such as robots autonomously completing wound suturing and excising diseased tissues in an open environment. The results of these research efforts have important guiding value for this study. In 2020, Pedram et al. [14] proposed a surgical robot autonomous suturing framework. This framework encompasses a needle-path planner, a pose estimator, and a controller. It cannot only achieve high-precision automatic suturing, but also has certain learning and self-adaptation capabilities, and can continuously optimize the operation strategy. Osa et al., from the University of Tokyo [15,16], proposed a method based on imitation learning and deep reinforcement learning to achieve the autonomous winding operation of surgical sutures. Similarly, Murali et al. [17] proposed a task-parameterized learning method. This method helps robots to flexibly adjust their strategies during excision operations by constructing a learning model that can adapt to different excision tasks. However, there is still a lack of research on the autonomous operation of arthroscopic surgical robots.

In this study, we developed an arthroscopic robotic system for meniscoplasty with autonomous operation ability. The main contributions of this paper are as follows:

- An arthroscopic robotic system was constructed. This system consists of two UR5 robotic arms, an RGB-D camera, and a sawbones knee joint model. Meanwhile, the end of the robotic arm is equipped with customized surgical instruments for meniscoplasty.
- A new two-stage cross-modal point cloud registration framework is proposed. Precise preoperative–intraoperative 3D point cloud alignment is achieved by fusing the Super4PCS algorithm with the improved ICP algorithm.
- A set of local autonomous motion-planning frameworks for robotic implementation is developed. We optimized the RRT path-planning algorithm to ensure that the paths planned by the algorithm can maintain the crescent shape of the meniscus. In addition, this study introduced the Remote Center of Motion constraints to enhance surgical safety.

The structure of the remaining part of this paper is arranged as follows. Section 2 elaborates on the system setup of the arthroscopic robotic system and related image registration and motion-planning algorithms. Section 3 presents the results of pre- and intraoperative point cloud registration and robot autonomous operation experiments. Finally, Section 4 summarizes the conclusions of this study.

## 2. Materials and Methods

### 2.1. System Setup

**Figure 1:**
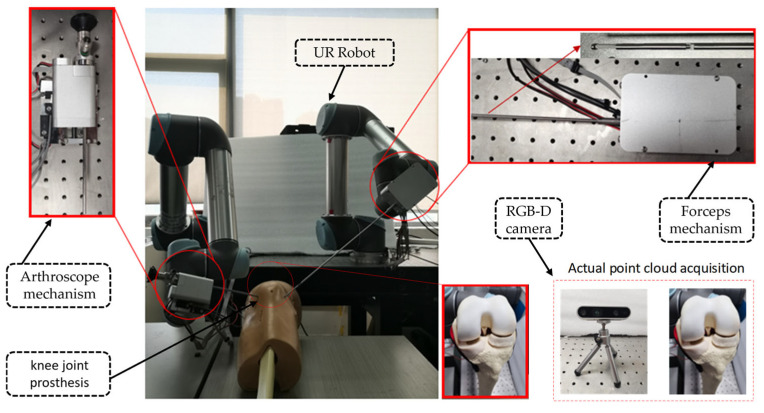
The arthroscopic robotic system setup.

The arthroscopic robotic system comprises four main components, as follows: two robotic manipulators (UR5, Universal Robots, Odense, Denmark), an RGB-D camera (RealSense D435i, Intel, Santa Clara, CA, USA), a knee joint prosthesis (SKU:1413-20, Sawbones, Vashon Island, WA, USA), and customized surgical instruments (forceps mechanism and arthroscope mechanism), as shown in Figure 1. Point cloud processing and robot control algorithms are developed by C++ in QT 5.12.0 and PCL on an Intel Core i7-9750 2.60-GHZ PC with 32-GB RAM. Through the TCP/IP communication protocol, the robot is connected to the PC, and through the SDK of the UR5 robot, it is possible to read the position of the end of the robot as well as the joint velocities of each joint in the Cartesian coordinate system.

In addition, we initially planned to use a binocular endoscope to acquire intraoperative 3D point clouds of the meniscus. However, due to its high cost, we substituted it with an RGB-D camera for point cloud image acquisition.

### 2.2. Customized Surgical Instruments

Aiming at the problems existing in current arthroscopic robots, such as the adjustment of instrument poses relying on the overall pose adjustment of the robotic arm, which leads to difficulty in achieving fine adjustment of the forward and backward displacement and rotation angle of the instruments, this study designed a customized forceps mechanism and an arthroscope mechanism for arthroscopic surgery. Both mechanisms possess two independent degrees of freedom, namely rotation and feeding.

**Figure 2:**
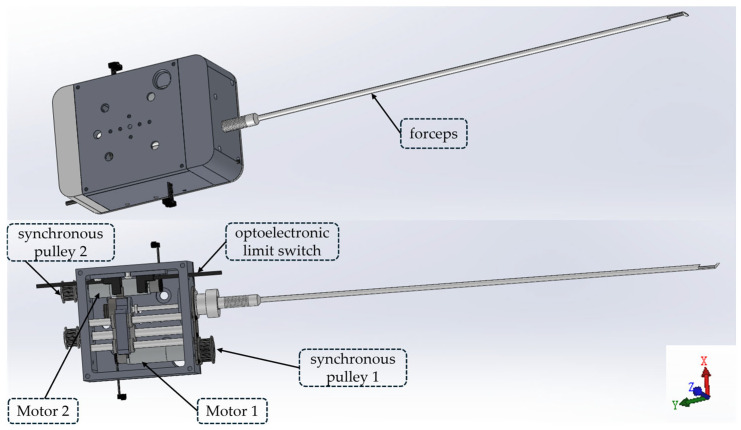
Structural diagrams of the forceps mechanism.

Firstly, this study referred to the design of bone forceps in clinical surgeries. By means of the feeding motion of the head of the forceps, the forceps mouth is made to bite, thus achieving the purpose of clamping and removing the damaged meniscus. The forceps mechanism is composed of the following three parts: the forceps, the forceps box module, and the robotic arm connection flange. Its overall structure is shown in Figure 2. The orientation referring to the coordinate system shown is in Figure 2, and the maximum dimensions of the forceps mechanism in its X, Y, and Z directions are 80 mm, 472.5 mm, and 44 mm, respectively. It can independently achieve two degrees of freedom, that is, rotation around the axis of the straight rod of the forceps and feeding along the axis of the straight rod of the forceps. The 360° rotation degree of freedom of the forceps is realized through the synchronous pulley 1 driven by the motor 1. The feeding motion of the forceps is realized through the synchronous pulley 2 driven by the motor 2, and its stroke range is from −1 mm to +14 mm. Specifically, the driven end of the synchronous pulley is connected to the ball screw. The rotation of the ball screw prompts the flange on it to generate axial displacement. When the flange moves forward, it pushes the inner rod of the forceps to move forward, and at the same time compresses the spring, making the forceps bite tightly. When the flange moves backward, the spring recovers from the compressed state to the extended state, which in turn drives the inner rod of the forceps to move backward, realizing the opening action of the forceps. Meanwhile, this study installed an optoelectronic limit switch, which is used to limit the positive and negative displacements of the forceps to prevent structural damage caused by over-travel.

**Figure 3:**
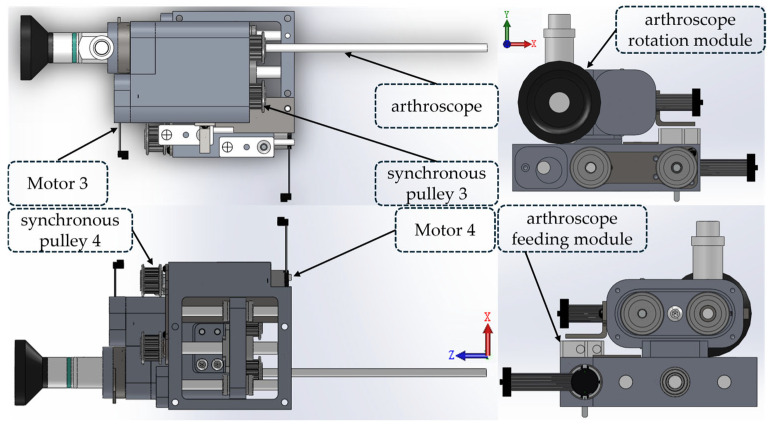
Structural diagrams of arthroscope mechanisms.

The arthroscope mechanism is composed of the following three parts: the arthroscope feeding module, the arthroscope rotation module, and the robotic arm connection flange. Its overall structure is shown in Figure 3. The orientation referring to the coordinate system is shown in Figure 3, and the maximum dimensions of the arthroscopic mechanism in its X, Y, and Z directions are 72 mm, 76.3 mm, and 230.4 mm, respectively. It can independently achieve two degrees of freedom, namely the rotation around the axis of the arthroscope and the feeding along the axis of the arthroscope. The motor 3 in the arthroscope rotation module drives the corresponding synchronous pulley 3 for transmission, thereby achieving the rotation degree of freedom of the arthroscope. Its rotation stroke ranges from −45° to 180°. The motor 4 in the arthroscope feeding module drives the corresponding synchronous pulley 4 for transmission, thus achieving the feeding degree of freedom. Its feeding stroke ranges from −1 mm to 14 mm. Specifically, the driven end of the synchronous belt is connected to the ball screw. The rotation of the ball screw prompts the flange on it to generate axial displacement. Because the arthroscope rotation module is connected to the flat plate of flange by bolt, the arthroscope rotation module will move axially along with the flange, further achieving the feeding degree of freedom of the arthroscope. This study installed an induction piece of the proximity switch on the arthroscope rotation module and a proximity switch on the arthroscope feeding module. These two components cooperate with each other to jointly limit the positive and negative displacements of the arthroscope in order to prevent the structural damage caused by the movement exceeding the stroke.

Considering control of the robot’s volume and weight, in the above-mentioned forceps mechanism and arthroscope mechanism, this study uses the DCX12L brushless DC motor produced by Maxon Group (Sachseln, Switzerland) as the driving device, and it is equipped with a GPX12 A-type gear reducer with a reduction ratio of 21:1 to reduce rotational speed and increase the torque. This study uses the ENX16 EASY 512IMP incremental encoder to obtain position information of the motor in real-time. The EPOS4 Compact 24/1.5 CAN motor driver produced by Maxon Company was selected to control the movement of the driving motors in the arthroscopic mechanism and the bite forceps mechanism. Communication between the computer and the driver is carried out through the Ixxat USB-to-CAN v2 CAN(HMS Networks, Halmstad, Sweden) card.

### 2.3. Two-Stage Cross-Modal Point Cloud Registration

#### 2.3.1. Coarse Registration Based on the Super4PCS Algorithm

This study performed a CT scan on the sawbones human knee joint model and obtained 650 CT slices. This study carried out a three-dimensional reconstruction of the knee joint model based on the marching cubes algorithm. Subsequently, the 3D Slicer software 5.8.0 was applied to perform a segmentation process on the reconstructed knee joint model, and the three-dimensional model of the meniscus was successfully obtained. To reduce the differences that may be caused by different types of modals, the meniscus models obtained before and during the operation in this study are all described by point cloud. To achieve fast and accurate registration, this study divides the point cloud registration process into the following two stages: coarse registration and fine registration.

**Figure 4:**
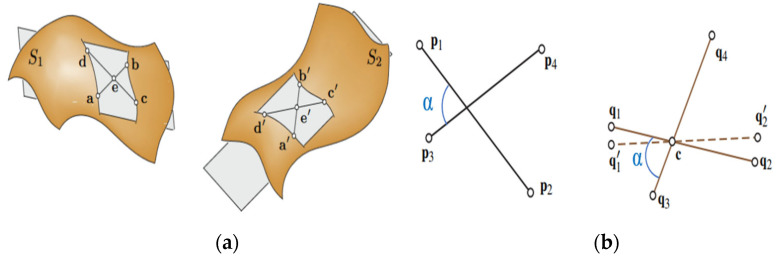
Schematic diagram of affine invariance. (**a**) Affine invariants; (**b**) add angular constraints.

Super4PCS (Super 4—Points Congruent Sets) is an efficient registration algorithm based on geometric consistency. Compared to traditional feature-based algorithms, such as the FPFH algorithm [18], the SHOT algorithm [19], the SIFT algorithm [20], and the SURF algorithm [21], it does not need to rely on local feature descriptors. Instead, it matches by calculating the geometric relationships between points, avoiding the complexity of descriptor calculation and matching. In addition, this algorithm has strong robustness against noise and partial occlusion and can complete the initial registration in a short time. The core idea of this algorithm is to utilize the consistency of affine invariants in a two-dimensional plane to construct corresponding relationships. As shown in Figure 4a, in the plane $S_{1}$, if any three points in the point set {a,b,c,d} are non-collinear, the lines of two different pairs of points will intersect at a point, denoted as the intersection point e. Based on this, two proportional relationships can be determined, as follows:

(1) $$r_{1}=\frac{\left\|a-e\right\|}{\left\|a-b\right\|}$$

(2) $$r_{2}=\frac{\left\|c-e\right\|}{\left\|c-d\right\|}$$

The ratios $r_{1}$ and $r_{2}$ are affine invariants. Based on this proportional relationship, the correspondence between the four-point set $\left\{a^{\prime },b^{\prime },c^{\prime },d^{\prime }\right\}$ in the plane $S_{2}$ and the four-point set {a,b,c,d} in the plane $S_{1}$ can be determined. However, a large amount of redundancy occurs when searching for the corresponding four-point set. As shown in Figure 4b, the four-point set $\left\{p_{1},p_{2},p_{3},p_{4}\right\}$ on the left corresponds to the four-point set $\left\{q_{1},q_{2},q_{3},q_{4}\right\}$ on the right, while the non-congruent set $\left\{q_{1}^{\prime },q_{2}^{\prime },q_{3},q_{4}\right\}$ also has affine invariance. Such a set that is affine invariant but inconsistent will significantly reduce the efficiency of the algorithm. Therefore, considering that, in a rigid transformation, the angle between two intersecting point pairs remains unchanged, the Super4PCS algorithm introduces an angle constraint. By determining the point set that matches the angle α of the base point set in the source point cloud, the search range of the congruent four-point set is effectively narrowed. In this process, the Super4PCS algorithm eliminates redundant candidate sets, significantly improving the speed of the algorithm.

The process of the Super4PCS algorithm for determining the corresponding four-point set is as follows. For a three-dimensional point cloud, firstly, according to the wide-area basis principle, randomly select three non-collinear points from the source point cloud P. Then, determine the fourth point to make it approximately coplanar with these three points, thus forming the base point set B={a,b,c,d}. In the target point cloud Q, based on the calculated proportional relationship, search for the four-point set that matches the base point set B, and its constraint conditions can be expressed as follows:

(3) $$e_{1}=q_{1}+r_{1}\left(q_{2}-q_{1}\right)$$

(4) $$e_{2}=q_{1}+r_{2}\left(q_{2}-q_{1}\right)$$

**Figure 5:**
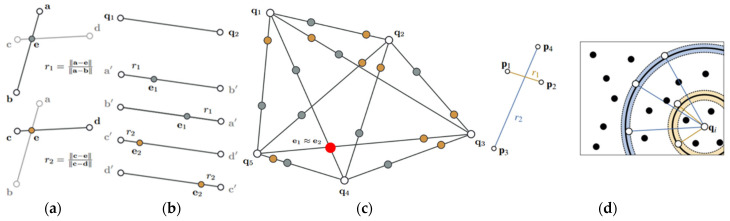
Search and extraction of congruent four-point sets. (**a**) Corresponding point set; (**b**) calculate the intermediate point; (**c**) search for the corresponding four-point set; (**d**) search for the corresponding point pairs.

As shown in Figure 5b, for the two intersecting point pairs in the target point cloud Q, calculate their intersection points according to the constraint conditions. For two line segments, if any point, $e_{1}$, in the first line segment coincides with any point, $e_{2}$, in the second line segment, or the distance between the two points is extremely small, then it can be considered that the four endpoints of these two line segments may be the corresponding points of the four points in the source point cloud, as shown in Figure 5c. Among them, the two sets of point pairs where the red intersection points are located are the four-point sets that match the base point set in the source point cloud. Based on this, the corresponding relationship of the four-point sets is determined, as shown in Figure 5a.

However, when the scale of the point cloud is large, the computational amount of point pair extraction will significantly increase. To conclude, the Super4PCS algorithm employs an efficient hyper-sphere rasterization technique, as shown in Figure 5d, for a set of four-point set $\left\{p_{1},p_{2},p_{3},p_{4}\right\}$ in the source point cloud P. Let $q_{i}$ be any point in the target point cloud Q. $r_{1}$ represents the distances between $p_{1}$ and $p_{2}$, and $r_{2}$ represents the distances between $p_{3}$ and $p_{4}$. δ is the tolerance set considering the noise in the data. Search for the spheres centered at $q_{i}$ with radii of $r_{1}\pm \delta$ and $r_{2}\pm \delta$, respectively, and filter out the meaningful point pairs. Thus, the computational cost is significantly reduced.

During the process of performing rough registration using the Super4PCS algorithm, this study found that due to the different acquisition sources of the two point cloud models, there is a certain difference in scale between the two point clouds. To reduce the impact of the scale difference on the registration accuracy, this study adopted the AABB bounding box algorithm [22] to conduct an initial scale estimation of the source point cloud and the target point cloud.

Based on the AABB bounding box algorithm, a cube is calculated. According to the vertex coordinates of the bounding box, the diagonal length is calculated, and then the ratio between the two point clouds is obtained to complete the calculation of the initial scale factor. Subsequently, this scale factor is applied to the point cloud to make the scales of the source point cloud and the target point cloud roughly consistent. Rough registration is performed on the point cloud with unified scales, and an initial homogeneous transformation matrix is obtained. The initial homogeneous transformation matrix and the initial scale factor are used as the original parameters in the fine registration process.

#### 2.3.2. Fine Registration Based on the Improved ICP Algorithm

When dealing with the registration of point clouds with inconsistent scales, a scale factor can be introduced into the ICP algorithm [23,24]. When solving the least squares problem in each iteration of the ICP algorithm, introducing a scale factor can effectively handle the scale differences and improve the registration accuracy.

Like the ICP algorithm, first determine two sets of mutually matching point sets. Suppose the source point set is $X=\left\{x_{1},x_{2},...,x_{N}\right\}$ and the target point set is $Y=\left\{y_{1},y_{2},...,y_{N}\right\}$. Define the scale factor s, the rotation matrix R, and the translation vector t. On this basis, define the error term of the i-th pair of points, as shown in Equation (5), as follows:

(5) $$e_{i}=sRx_{i}+t-y_{i}$$

Construct a least squares problem. The scale factor s, the rotation matrix R, and the translation vector t that make the sum of the squared errors in Equation (6) reach a minimum value, and they are the parameter values we seek in the following equation:

(6) $$\underset{R,t,s}{\min}\frac{1}{2}\sum_{i=1}^{N}{\left\|sRx_{i}+t-y_{i}\right\|}_{2}^{2}$$

To avoid the obtained scale factor s being too large or too small, this study sets a threshold for it. Let the diagonal length of the AABB bounding box of the source point cloud be $L_{1}$, and the diagonal length of the AABB bounding box of the target point cloud be $L_{2}$. Denote $a=0.8\frac{L_{2}}{L_{1}}$ and $b=1.2\frac{L_{2}}{L_{1}}$. Then, the value range of λ is as follows:

(7) $$s=\left\{\begin{array}{l} a\;s\leq a\\ b\;s\geq b\\ \frac{\sum_{i=1}^{N}\langle Rx_{i}^{'},y_{i}^{'}\rangle }{\sum_{i=1}^{N}\langle x_{i}^{\prime },x_{i}^{\prime }\rangle }\;a<s<b\; \end{array}\right.$$

Substitute the value of the scale factor s into the least squares problem and calculate the rotation matrix R by performing singular value decomposition on the covariance matrix. After obtaining the rotation matrix R and the scale factor s, further solve for the translation vector t. Apply the obtained rotation matrix R, scale factor s, and translation vector t to the source point cloud, and then enter the next iteration process. Until the error variation between two adjacent iterations is less than the set threshold, or the number of iterations exceeds the maximum number of iterations, exit the iteration, complete the fine registration of the point cloud, and obtain the final homogeneous transformation matrix.

**Figure 6:**
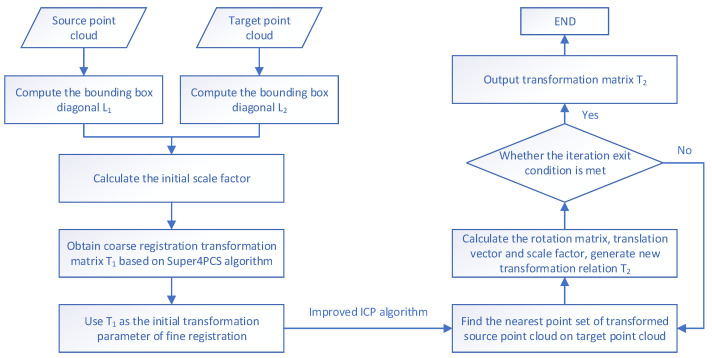
Flowchart of the registration framework.

In conclusion, the two-stage transmembrane state point cloud registration framework proposed in this study includes rough registration based on the Super4PCS algorithm and fine registration based on the improved Iterative Closest Point (ICP) algorithm. The process is shown in Figure 6.

### 2.4. Motion-Planning for Autonomous Operation

The autonomous motion framework of the robot is as follows: First, set the starting point and the ending point of the path. Then, the robot automatically plans a path and calculates the corresponding motor drive amount to control the surgical instrument to complete the meniscus resection. To improve surgical safety, the RCM motion constraint is introduced during the robot’s motion process. Since the motor control part is relatively simple, this section mainly introduces the RCM algorithm and the path-planning algorithm.

#### 2.4.1. Path-Planning Based on the Improved RRT Algorithm

After the doctor determines the starting and ending positions of the meniscus resection by observing the patient’s CT images, the robot automatically plans a feasible path between these two points and uses each planned path point as the target position for the robot to remove the damaged meniscus each time. The Rapidly Exploring Random Tree (RRT) algorithm [25] is a commonly used path-planning algorithm, which demonstrates good adaptability in a high-dimensional and complex space environment. This study will make improvements based on the RRT algorithm and design a reasonable cost function to ensure that the path autonomously planned by the robot can meet specific shape requirements, thus conforming to the actual clinical needs.

Firstly, the voxel meshing method is applied to set up the obstacle space. After dividing the point cloud into mesh blocks, the voxels that do not contain points are screened out as obstacles, and the remaining space is regarded as free space.

Considering the sparse characteristics of the point cloud, during each random sampling process, a tolerance value is introduced to enable the path points to be distributed within a relatively small range around the point cloud. This is because the surgical instrument can perform the resection operation on the damaged meniscus in the space near the target point, so the path points do not need to be strictly within the point cloud. At the same time, this can also prevent the sampling process from falling into an infinite loop due to the inability to find suitable points.

**Figure 7:**
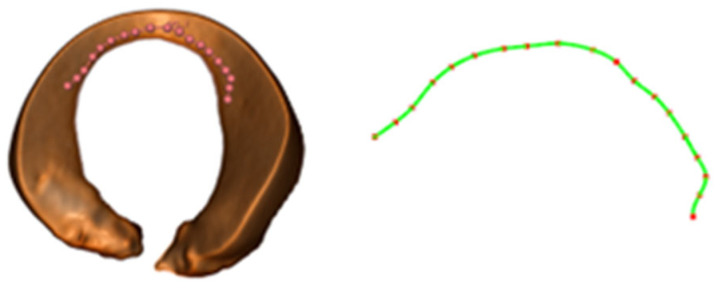
Schematic diagram of the fitting curve.

To maintain the crescent shape of the remaining meniscus, this study introduces a cost function into the RRT algorithm to evaluate each newly generated node so that the path points can not only meet the basic connection conditions, but also select more appropriate paths according to the cost. To this end, according to the crescent shape of the edge of the complete meniscus, a curve function is fitted as a reference. With the help of the 3D Slicer software, a series of points on the edge of the meniscus model are selected, and then the coordinates of these points are exported. The B-Spline algorithm [26] is used for the fitting operation, thereby generating the actual curve equation. To achieve a better balance among smoothness, control ability, and computational efficiency, the order is set to the third order. The resulting fitted curve is shown in Figure 7.

After obtaining the fitted curve, the shape matching cost function is defined. By calculating the curvature, it is determined whether the shape of the path is consistent with the trend of the fitted curve, and the curvature difference between the path segment and the corresponding segment of the fitted curve is taken as the cost. Suppose the parametric equation of the fitted curve is $C\left(u\right)$, the current path point set is $\left\{P_{0},P_{1},\ldots ,P_{n}\right\}$, and the number of current path points is *n*. Let the curvature of the fitted curve at the parameter $u_{i}$ be $\kappa \left(C\left(u_{i}\right)\right)$, and the curvature of the path segment at the path point $P_{i}$ be $\kappa \left(P_{i}\right)$. On this basis, the cost function is defined as follows:

(8) $$\cos t=\frac{1}{n}\sum_{i=1}^{n-1}\left|\kappa \left(P_{i}\right)-\kappa \left(C\left(u_{i}\right)\right)\right|$$

Suppose the three adjacent points on the path are $P_{i-1}$, $P_{i}$, and $P_{i+1}$, respectively. Then, the curvature $\kappa \left(P_{i}\right)$ of the path at the point $P_{i}$ can be approximately expressed as follows:

(9) $$\kappa \left(P_{i}\right)=\frac{\left\|\left(P_{i}-P_{i-1}\right)\times \left(P_{i+1}-P_{i}\right)\right\|}{\left\|P_{i}-P_{i-1}\right\|\cdot \left\|P_{i+1}-P_{i}\right\|}$$

Suppose that the tangent vector of the fitted curve at the parameter u is $C^{\prime }\left(u\right)$ and the acceleration vector is $C^{\prime \prime }\left(u\right)$. Then, the curvature $\kappa \left(C\left(u_{i}\right)\right)$ of the curve at u is defined as follows:

(10) $$\kappa \left(C\left(u_{i}\right)\right)=\frac{\left\|C^{\prime }\left(u\right)\times C^{\prime \prime }\left(u\right)\right\|}{{\left\|C^{\prime }\left(u\right)\right\|}^{3}}$$

During the initialization stage, a cost threshold $\cos t_{threshold}$ is set. Whenever a new node is generated, calculate the cos⁡t of the newly generated path segment. If this cost is less than the set threshold, it is determined that the shape of the path is consistent with the trend of the target curve. Add this node to the expansion tree and incorporate the new path segment into the connection relationship of the tree. If this cost exceeds the set threshold, discard the path and perform a resampling operation on the node. Finally, check the distance between the new path point and the target point. When this distance is small enough, it is determined that the path-planning is completed; then, extract the generated path from the expansion tree.

#### 2.4.2. Remote Center of Motion Control

Knee arthroscopic meniscoplasty requires making 2 to 3 small incisions at the knee joint. The surgical instruments enter the patient’s body through these small holes for the operation. Since the surgical instruments cannot move in a large range, this study introduces the Remote Center of Motion (RCM) constraint [27]. During the surgical process, the RCM point is set, and through the algorithm, it is ensured that the movement of the surgical instruments always passes through the RCM point, thus greatly reducing the risk of causing harm to the patient.

After introducing the RCM motion constraint, the pose of the robot when moving to the target point is restricted, while the position vector of the target point remains unchanged. Therefore, after introducing the RCM constraint, the problem essentially transforms into solving the rotation matrix used to represent the pose of the target point.

**Figure 8:**
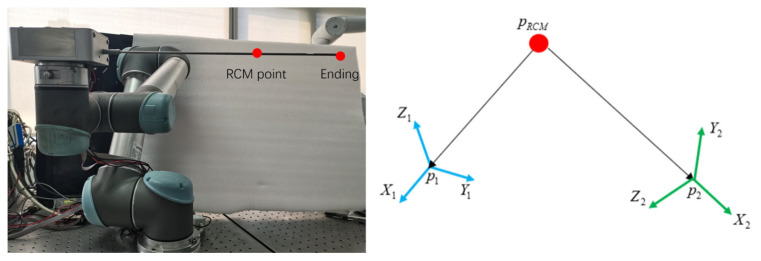
Solution of RCM (Remote Center of Motion) constraints.

As shown in Figure 8, assume that the Remote Center of Motion point is $p_{RCM}$, and the current position of the end of the instrument is $p_{1}$. Establish the coordinate system according to the current pose. Let the position of the target point be $p_{2}$; however, its pose is unknown, and the corresponding coordinate system cannot be determined either.

During the surgical process, the rod of the instruments will pass through $p_{RCM}$ all the time. Therefore, according to the coordinates of point $p_{RCM}$ and point $p_{2}$, the direction of the *X*-axis of the coordinate system $p_{2}$ can be determined as follows:

(11) $$x_{2}=norm\left(p_{2}-p_{RCM}\right)$$

In a three-dimensional space, the rotation of any rigid body can be decomposed into a rotation around a specific axis. This axis is called the rotation axis, and the rotation angle represents the amplitude of the rigid body’s rotation around this rotation axis. Regarding the directions of the *Y*-axis and *Z*-axis of the coordinate system $p_{2}$, they can be solved by using the concepts of the rotation axis and the rotation angle. Suppose the coordinate system $p_{2}$ is obtained by rotating the coordinate system $p_{1}$ around the rotation axis V by an angle θ. The rotation axis V and the rotation angle θ are determined through Equations (12) and (13), which are as follows:

(12) $$V=x_{1}\times x_{2}$$

(13) $$\theta =\arccos\left(x_{1}\cdot x_{2}\right)$$

Then, define the rotation matrix R to represent the transformation relationship after rotating around the rotation axis V by an angle θ. According to Rodrigues’ formula, the rotation matrix R can be expressed in the following form:

(14) $$R=\cos\theta \cdot I+\sin\theta \cdot skew\left(V\right)+\left(1-\cos\theta \right)V\cdot V^{T}$$

Among them, $skew\left(V\right)$ is the skew-symmetric matrix of the rotation axis. After obtaining the rotation matrix R, apply it to the coordinate system $p_{1}$, and the direction of the coordinate system $p_{2}$ can be obtained. Furthermore, the pose of the target point can be deduced. Based on the position vector of the target point and the rotation matrix R, the pose of the robot is determined. Then, according to the inverse kinematics principle, the angles of each rotating joint of the robotic arm are determined to realize the movement of the robot under the RCM constraint.

## 3. Results

Firstly, the arthroscopic robotic system was built. Subsequently, the point cloud registration and motion-planning algorithms for robotic autonomous operation was integrated into the software system of the robot. With the built robotic platform, the preoperative to intraoperative point cloud registration experiment and the robot autonomous operation experiment were conducted.

### 3.1. Pre- and Intraoperative Point Cloud Registration Experiment

**Figure 9:**
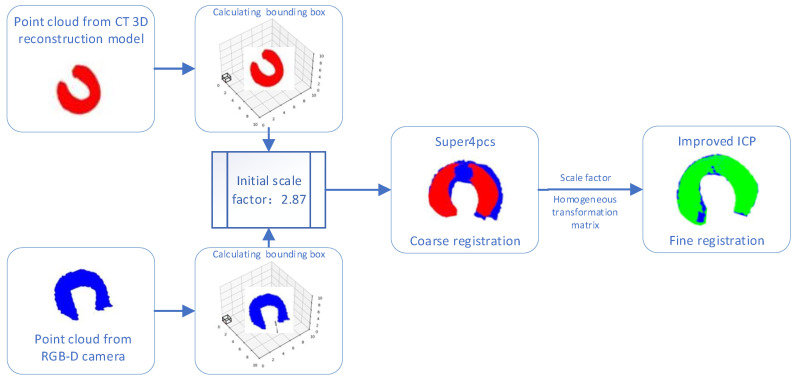
The procedure of registration experiment.

The process of the point cloud registration experiment is shown in Figure 9. The red point cloud is the meniscus point cloud model obtained by reconstructing CT images, and the blue point cloud is the point cloud model directly captured and extracted by the RGB-D camera RealSense D435i (Intel, Santa Clara, CA, USA). There is a large difference between the two point cloud models. First, calculate the initial scale factor and perform coarse registration. Set the red point cloud as the source point cloud and set the blue point cloud as the target point cloud. Solve the rough transformation relationship between the source point cloud and the target point cloud. After calculation, the coarse registration transformation matrix $T_{1}$ is obtained, and the initial scale factor is 2.87042. The result of the coarse registration is shown in Figure 9. The two point clouds are roughly aligned.

Next, perform fine registration. Use the result of the coarse registration as the initial value of the fine registration and set the number of iterations to 30. The obtained transformation matrix is $T_{2}$, and the obtained scale factor is 1.06347. The result of the fine registration is shown in Figure 9, where the green point cloud represents the source point cloud and the blue point cloud represents the target point cloud. The difference between the point clouds is represented by the Mean Squared Error (MSE), and the calculation method is shown in Equation (15), as follows:

(15) $$e=\frac{1}{N}\sum_{i=1}^{N}{\left\|sRx_{i}+t-y_{i}\right\|}^{2}$$

After calculation, the MSE value of the coarse registration is 5.74276, and the MSE value after the fine registration is reduced to 0.19641, which meets the accuracy requirements of medical applications.

### 3.2. The Experiment of Motion-Planning and Robot Autonomous Operation

To validate the effectiveness of the robot autonomous operation framework, this study simulated the scenario of meniscoplasty for a small radial tear in the middle part of the meniscus and conducted an experiment on motion-planning and robot autonomous operation on sawbones’ lower-limb prosthesis.

First, the initialization parameter setting work was carried out. Based on the lesion information presented in the three-dimensional point cloud of the meniscus, the target area, the starting point and the ending point of the path were determined. The target area was required to completely cover the meniscus point cloud. Its coordinated range was set as 50 mm≤x≤60 mm, 40 mm≤y≤70 mm, and 30 mm≤z≤70 mm. The starting point coordinates were set as $\left(53.0\;\mathrm{mm},48.0\;\mathrm{mm},61.0\;\mathrm{mm}\right)$, and the ending point coordinates were set as $\left(54.0\;\mathrm{mm},53.0\;\mathrm{mm},36.0\;\mathrm{mm}\right)$. Subsequently, the B-Spline algorithm was applied to fit the crescent-shaped contour in the middle of the meniscus to obtain the reference curve for the cost function in the RRT algorithm.

After the initialization parameter setting was completed, path-planning work was carried out based on the improved RRT algorithm. The obtained planned path contained 39 path points. The coordinates of the above-mentioned path points were all based on the CT coordinate system. By means of the obtained homogeneous transformation matrix of the CT coordinate system relative to the robot base coordinate system, the coordinates of path points were transformed to the robot base coordinate system, thereby obtaining the target points in the robot autonomous surgical process.

**Figure 10:**
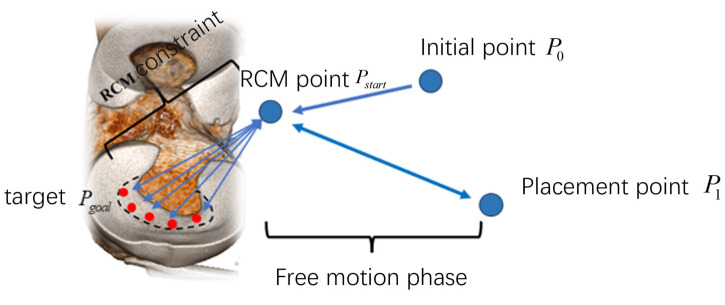
Robot autonomous operation framework.

**Figure 11:**
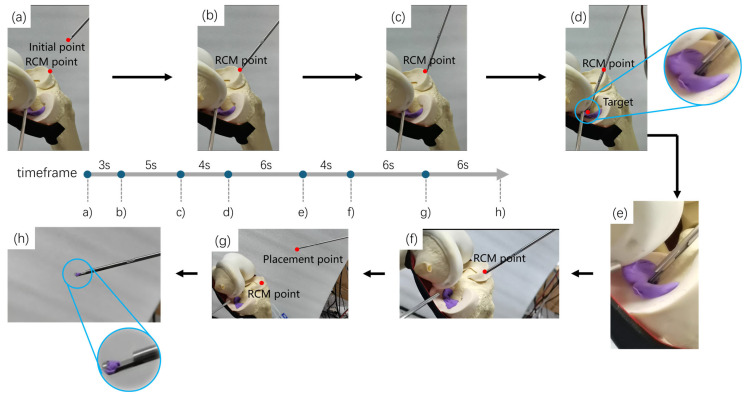
The experiment flow of robot autonomous operation. The process is as follows: (**a**,**b**) The robot moves freely to the RCM point; (**b**,**c**) The robot adjusts its pose under the constraint of RCM; (**c**,**d**) The robot moves to the target point; (**d**,**e**) The robot excises the damaged meniscus; (**e**,**f**) The robot retreats to the RCM point; (**f**,**g**) The robot moves to the position for placing the meniscus; (**g**,**h**) The forceps release and place the meniscus.

Based on the designed robot autonomous operation framework, as shown in Figure 10, an experiment of the robot autonomously excising part of the meniscus was carried out. The coordinates of the RCM point were set as $\left(95.808\;\mathrm{mm},-472.068\;\mathrm{mm},393.909\;\mathrm{mm}\right)$, and the coordinates of the initial placement point of the damaged meniscus were set as $\left(-24.705\;\mathrm{mm},-436.175\;\mathrm{mm},440.675\;\mathrm{mm}\right)$. The target point of each excision process was the pre-planned path point. Given the large number of target points, only the process of the robot autonomously moving to one of the target points to excise the damaged meniscus is shown here, as shown in Figure 11, which takes about 34 s. In Figure 11, (b) denotes the robot free motion phase to move from the initial positioning point to the RCM point in the *MoveL* mode of a UR robotic arm; (c) and (d) denote the movement of the robot from the RCM point to the target point under RCM constraints, with the process of (b) to (c) as the robot adjusting its posture around the RCM point, and the process of (c) to (d) as the robot moving along the axial direction of the end instrument to the target point; (e) denotes the driving of the motor drives the bite forceps to bite off the broken meniscus; (f) indicates the robot moves from the target point to the RCM point under the RCM constraint; (g) indicates the robot’s free-motion phase, which moves from the RCM point to the broken meniscus placing point in the *MoveL* mode of the UR robotic arm; and (h) indicates that the motor goes back to zero so that the bite forceps opens and the bitten-off broken meniscus will fall into the placing tray. At this point, the process of one resection point is finished. Next, the program will update the position of the next target point, then the robot will move to the RCM point in a free motion mode to carry out the next resection process until it goes through all target points, at which point the robot autonomy operation is finished and the program is exited.

Experimental results show that the path planned based on the improved RRT algorithm completely covers all damaged meniscus areas and maximally preserves the crescent-shaped morphology of the meniscus. The robot efficiently implemented the meniscoplasty operation according to the autonomous motion-planning framework.

## 4. Conclusions and Discussion

This study successfully developed a novel arthroscopic robotic system with autonomous surgical capabilities, demonstrating its efficacy in automatically performing torn meniscus resection during meniscoplasty. The robotic system integrates a customized 2-DOF arthroscopic module and bite forceps module at its end. To bridge the gap between preoperative 3D point cloud data and the physical surgical space, a two-stage cross-modal point cloud registration framework was proposed. Leveraging the Super4PCS algorithm for initial alignment and an improved Iterative Closest Point (ICP) algorithm for fine-tuning, this framework achieved an impressive registration accuracy with a mean squared error (MSE) of 0.19641. Furthermore, a comprehensive motion-planning and autonomous operation framework was devised. The Rapidly Exploring Random Tree (RRT) path-planning algorithm was enhanced by incorporating a curvature difference cost function, ensuring path compliance with predefined geometric constraints. Additionally, the Remote Center of Motion (RCM) principle was integrated into the robotic autonomous movement, enhancing surgical safety. Experimental validation conducted on a sawbones knee joint prosthesis successfully verified the feasibility and stability of the autonomous operation capabilities of the proposed arthroscopic robotic system.

However, there are still some deficiencies in this arthroscopic robotic system that need to be improved. Currently, in the process of path-planning, we adopt a dense sampling strategy to ensure the thoroughness of meniscus resection, which leads to many redundant target points and a relatively low efficiency of autonomous operation of the robot. In our future work, we will consider introducing a dynamic resection area evaluation model, optimizing the interval and distribution of path points, and improving the efficiency of autonomous operation.

## Data Availability

Data are contained within the article.
